# French Red Cross Ladies in International or Universal Exhibitions (1867–1937)

**DOI:** 10.3389/fsoc.2019.00054

**Published:** 2019-07-10

**Authors:** Corinne M. Belliard

**Affiliations:** Sorbonne Nouvelle, Université Paris-Sorbonne, Paris, France

**Keywords:** red cross, women, societies, world fairs, France

## Abstract

This paper attempts to pinpoint what French Red-Cross women were doing in the international exhibitions from 1867 to 1937. They engaged their energies into organizing meetings, exhibitions, and into healing, receiving awards for their work. In spite of their dual activity of exhibitors and healthcarers, they had no specific pavilion at the world fairs. They hovered between the worlds of politics and medicine as distinguished guests but without their own space.

Although at the turn of the twentieth century, it was probably not surprising to notice the presence of women within the Red Cross Societies and in International Exhibitions, belonging to both institutions had not yet been considered. At first sight, they seemed both to be and not to be settled in a global environment. No iconographies represent Red Cross ladies at work in International Exhibitions. Yet, they were on Parisian sites starting from the early phase of the 1867 Universal Exhibition up until the 1937 International World Fair, the last paradigm of its kind. An intuitive thought places women from Red Cross Societies on the rescuing side in the public spaces of exhibitions all the way through this period.

The issue here, other than knowing who they were, is to identify what, in the eyes of the world, they did: were they just ordinary members of nationwide societies, professional health staff who ensured the protection of visitors in international exhibitions? Or, did they carry out, in a national agenda, an International philanthropic activity, showing generosity, kindness, benevolence, help, assistance and “love of mankind” (Littré, 1982)? In other words, were they local representatives casting transnational objectives at world fairs? As “regulars” in congresses and International Health Committees, did women in French Red Cross Societies speak with one voice in International Exhibitions? Was there a prevalent society of women at the world fairs? To what extent did International Exhibitions exhort women in French Relief societies?

## Sharing Ideals

The involvement of French Red Cross women in International Exhibitions is an aftermath of Solférino. It brings us back to the emotion Henry Dunant (1828–1910) a philanthropist (Jaeger, 2009)^1^ from Geneva expressed the day after the Battle of Solforino (Duquet, 1896). Following the conflict for Italian unity with Franco-Piedmontese troops who were opposing the Austrians, in 1859, Dunant could not forget the hundreds of wounded piled up on the battlefield. He wrote about it to the Countess de Gasparin (1813–1894) to beg her “to start a fund for relief or at least to collect money in Geneva” (Française, 1974, p. 47). At the same time, Dunant noticed that Italian women were spontaneously helping and comforting. He particularly recalled that:

“In the church the women of Lombardy go from one to the other with jars and pitchers full of water, which serve to appease the thirst and to bathe the wounded. Some of these improvised nurses are good-hearted old women, other are charming young girls. Their gentleness, goodness, compassion and their attentive care restored a little courage to the wounded” (Dunant, 1911, p. 32–33).

The Countess Verri-Borromeo (-1860) took the lead of the Italian Ladies Relief Committee (Sturzenegger, 1914, p. 12). In providing moral support and assistance in war times, women both soothed the pain and shared the same drive.

“Women of Castiglione, seeing that [Dunant made] no distinction in nationality, imitate[d] [his] example, showing the same kindness to all these men of such different origins and who [were] to them all equally strangers” (Dunant, 1911, p. 40).

They developed the assistance that existed from the past^2^. Women's “uncalculated acts and behavior”^3^ inspired Dunant who recorded them in his memories (Dunant, 1862). But his writing did not bring him relief. He never showed any “resilience” (Moynier, 1903, p. 4–5) and he co-founded with Gustave Moynier^4^ (1826–1863) a relief society in 1864. His impulse also came from 16 plenipotentiaries^5^ who met at the Geneva Convention and whose nations engaged that same year in treating the wounded, the sick, and respecting medical personnel (see also Maxime Du Camp, 1889, p. 75–98; Moynier, 1903, p. 27; French Red Cross, 1936, p. 15). In military camps a flag and a white arm-band with a red cross was adopted as the emblem. But this convention did not remain just a simple moral legislation, “an affair for the government” (Werner, 1938, p. 42–43).

Both doctors and women felt for their fellowmen and put a lot of effort into curing and caring. In France, the *Société de Secours aux Blessés Militaires* (S.S.B.M.—Society for the Relief of Wounded Soldiers—) started with this motto “Inter Arma Caritas” (In War, Charity). Its main objective was to assist civilians and military who were wounded. Its members, having Catholic and Royalist backgrounds,

“…were to help not only their own country's sick or wounded, but also ‘all the unfortunate who fall into their hands, irrespective of nationality.’ This mutuality would ensure proper treatment for any wounded man which ever society had him in its care” (Boissier, 1985, p. 336).

S.S.B.M. ladies were generally acted in the name of a community, a country or a nation participating in the birth of the word nationality (Noiriel, 1995) whose base was religious at the very beginning. Ordinary S.S.B.M. female members did not however make a name in this International philanthropic activity. That would have been sounded since women's organizations were developing beyond the S.S.B.M. *La France Charitable et Prévoyante* for example recorded 53 societies founded by women^6^.

S.S.B.M ladies had therefore predictably some figureheads such as the Grand Duchess Louise de Bade (1838–1923) was to be remembered for praising the Marquis de Vogüé, President of the French Red Cross Central Committee, and Gustave Moynier, President of the International Red-Cross Committee, who wanted to extend “charitable activities all over the world” (Boissier, 1985, p. 351). Chairwomen of the S.S.B.M. Ladies Committee also went down to history, especially when it was reset in 1875 (Pineau, 2006, p. 13). Madame la Maréchale Niel was then less known for her Red Cross philanthropic activity than her followers the Comtesse de Flavigny, the Princesse Czartoriska, Madame la Maréchale de Mac-Mahon, the Duchesse de Reggio, the Countesse Haussonville, the Marquise de Montebello, Madame la Maréchale Lyautey, and Mademoiselle d'Haussonville^7^. Madame la Maréchale Lyauté (1862–1953) was particularly celebrated for her service in the Red-Cross. She was awarded the Légion d'Honneur because she “worked for the human body of France” (Chavenon, 2010, p. 207) whereas her husband worked to glorify the country. Their fame was set according to the traditional gender sphere of activity.

As the S.S.B.M. was gradually increasing in world fame, the second Universal Exhibition opened in Paris in 1867. The first of French Relief societies had long thought it would be an opportunity to assert itself in this International happening (Foucault, 1936, p. 27; see as well Pineau, 2014, p. 17). It was rather pleased to respond to the “daring”^8^ invitation of the organizer, Frederic Le Play (1806–1882). He wanted all existing societies and new circles to participate and collaborate. His idea was to offer the S.S.B.M. and the other 19 International Relief Societies a chance to put on display articles over 700 m^2^ (Foucault, 1936, p. 27). He also wished to “stimulate the zeal of academicians and industrials so as to improve rescue services which would play in favor of the wounded” (Moynier, 1901, p. 8–9). A large number of inquisitive visitors and members of relief societies would visit the Red Cross exhibition site. The S.S.B.M. staff specifically invited their visitors to pay tribute in front of the statue of H. Dunant's bust (Gigan, 1943, p. 191–192).

For the International Committee of the Red Cross (I.C.R.C.), Le Play's invitation was primarily the opportunity to promote their first International Conference^9^. It was also the way to notice how the S.S.B.M. would organize a meeting for International Red Cross societies “in a more peaceful setting” (Boissier, 1985, p. 334) other than on the battlefields. For Clara Barton (1821–1912), a lady in charge of the hospitals during the American Civil War and future founding mother of the American Red Cross (Price, 1954; see also Jones, 2013), this was the opportunity to highlight a central authority (Boissier, 1985, p. 340). As “it was believed that Red Cross societies had a common purpose … [and] should be united and interdependent” (Boissier, 1985, p. 333), the Parisian Universal Exhibition was the place to apply it. Le Play's invitation allowed therefore Red Cross societies to establish long lasting encounters and identify the objectives and expectations of the society's future International Conferences (Moynier, 1901, p. 12). In addition to this, the second Universal Exhibition bestowed the I.C.R.C. to produce a list of people who had given their services to the relief societies and who deserved a medal.

In willing to distribute awards, the I.C.R.C. was acting like the organizers of the 1867 Exhibition. It was also under pressure to take part in the world fair and displayed among 600 objects, some publications (Foucault, 1936, p. 27). But, these writings drew little interest from visitors. The panel of judges at the Universal Exhibition awarded nevertheless a Grand Prize to the S.S.B.M. Its intention was primarily to honor Henri Dunant, the man who healed wounded men thanks to the help of women. “Nobody then contested the right for the International Committee of the Red Cross to be awarded in this way” (Moynier, 1903, p. 33). In the eyes of the world, International Relief Societies benefited from the S.S.B.M.'s success when some women of the French Red Cross society decided to break apart the national unity.

## Competing with Other Relief Societies

By 1879 the S.S.B.M., which did particularly well during the 1870 war in managing hospitals and ambulances (Rouge, 1963, p. 16–17), underwent a period of division transpiring on the international scene. Some French female members started to be concerned with the management of the society left exclusively to men (Pineau, 2006, p. 13). Therefore, they splitted officially to found a new society, the *Association des Dames Françaises* (A.D.F.—French Women's Society—) that was solely managed by women (Association des Dames Françaises, 1900, p. 6). On the International scene, the A.D.F. was also particular in that it was composed of members with nursing skills (Rouge, 1963, p. 17, 36). The split with the S.S.B.M. was also triggered by the fact that Dr. Duchaussoy noticed that during the 1870 war, outstanding women distinguished themselves publically in spite of their lack of medical skills. He recorded then that women provided all the attention needed (Chevalier, 1986, p. 29). He felt that a “completely independent paramedic public school”(Pineau, 2006, p. 13) should be established and confer degrees to take care of mankind on the International arena. Therefore, he co-founded with Emma Koechlin Schwartz the A.D.F. (Pineau, 2006, p. 21). Exams were regularly organized in different A.D.F. local committees. The Nice Committee, for example, reported that:

“The exam had two parts. The first part was held at the Dispensary of the Ecole Pratique, where applicants had to do bandages on patients and answer questions about the material used in this school and the antiseptic precautions concerning the material. The second part of the exam was held at the Headquarters of the Committee, and dealt with questions on 1/anatomy and physiology, 2/hygiene, 3/pharmacy, 4/medicine”^10^.

On their application forms, applicants in every single committee were required to show evidence of their regular attendance at classes and be specific about their membership^11^. The A.D.F. chaired for nearly 30 years by the Countess Foucher de Careil (1881–1911), a wealthy lady who enjoyed the position of her husband as Senator and Ambassador in Austria^12^, had always spoken highly of its activities in prestigious and International circles. At the Paris 1900 world fair, the society took particularly great care in exhibiting therapeutic objects.

Boats with their life-saving appliances, medical dressings, and stretchers for transporting the wounded and sick after a naval battle were thought to have been amongst the first things displayed. The A.D.F. also considered deploying different models of stretchers which were dragged by bicycles or carts. This was a way for the society to show its International scope^13^. But, Maréchal Lyautey (1854–1934), the organizer of the 1900 Universal Exhibition, as regards the other societies, only granted them a small space. The A.D.F. was then obliged to abandon its initial plan. As a result, the society presented material that was lighter in weight, material such as hygienic spittoons, stretcher-bearers' bonk bags, canteen, and rescue bags, hospital bed-linen, bandages, plasters, but also hospital beds and cars to transport the sick and wounded. Furthermore, the A.D.F. decided to extend the world fair and exhibit outside the official venue, in its hospital in Auteuil, a hospital-tent, with all the material needed for 20 beds. The A.D.F. also invited guests in its general stores (Association des Dames Françaises, 1900, p. 13–14, 38).

As for the other relief society the S.S.B.M, it was satisfied with the space that was allotted to it in the pavilion for hygiene and sanitary equipment (Exposition Internationale, 1900, p. 254). That fitted with the little the society had to display, that is to say charts showing its local committees, temporary provincial hospitals, main county hospitals, sick-bays, hospital bed-linen, and bandages^14^. Even the latest of the relief societies, the *Union des Femmes de France* (U.F.F—French Women's Union—), founded in 1881 by Mme. Koechelin-Schwartz (1838–1911), was contented^15^. The A.D.F. held particularly strong on its choice to display as many objects as possible at the 1900 world fair because the I.C.R.C. rather wanted to interact with one French Red Cross society, and that was the S.S.B.M (Pineau, 2006, p. 13). The A.D.F. would not let the S.S.B.M. hold a world-wide dimension. Twenty-two years after the first International Congress for the Rights of Women (Auclert, 1878) and in the midst of a similar situation^16^, a society managed by women wanted to be more visible than a society with a mixed management. Red Cross ladies put their case forward in the 1900 International event but in a moderate way. Therefore, the A.D.F. expressed, in the *International Bulletin of Red Cross Societies*, the need to gather together all men of humanity and charity during the world exposition^17^. These subdued national tensions were unheard and most probably ignored by the 1900 exhibition organizers who finally decided to award the I.C.R.C. with a Grand Prize^18^. Women in French Red Cross Societies were inconspicuous although they exposed.

Faced with these different national components, problems were not to be smoothed at the 1931 International Colonial Exhibition. France's colonial empire was by then a driving force in women's presence at this particular World Fair. It provided the opportunity for French Red Cross ladies to participate into the country's world power. They were involved in a larger project carried out by men such as Jules Ferry (1832–1893) who openly stated “the higher races had duties over the lower races” (Ferry, 1885). Colonialism had revived completion between relief societies.

Yet, the I.C.R.C. wished that, among the competing French relief societies, only one would correspond with the well-known General Commissioner, the Maréchal Lyautey. Thus the I.C.R.C. set an inter-society commission to make a decision and called upon their “spirit of fraternity”^19^. But French Red Cross societies preferred to exhibit separately. Having “no power to influence the destiny of the Red Cross [Societies, the I.C.R.C.] stressed endlessly that the national societies enjoyed total independence” (Boissier, 1985, p. 335). “To voice its opinion, the [I.C.R.C only] had the Bulletin [and that was the] link between one society and another” (Boissier, 1985, p. 335).

Therefore, in 1931, some sort of common strategy, showing a “spirit of solidarity”^20^ was discussed. It was finally agreed that French Red Cross societies were going to exhibit both individually and together. A standard exhibition on the second floor of the Palace Museum gave insight into the constitutions and the relief societies' aims and means of action^21^. Flyers and leaflets completed these tables of activities^22^. Beside this event, the three Red Cross societies exhibited in the colony pavilions where they carried out their activities. The S.S.B.M displayed its constitution and also maps and photographs relating to past and present activities on boards in the Indochinese pavilion. The A.D.F. set up an identical arrangement in the Dakar and Guadeloupe pavilion. As for the U.F.F., it displayed in the Malagasy and Togolese pavilions small red crosses on a map of France, which showed where the society owned regional committees.

The 1931 International Colonial Exhibition not only more or less enabled the S.S.B.M., the A.D.F. and the U.F.F. to speak with different voices, but also to shed light on the U.F.F. fiftieth anniversary^23^. The relief society was invited to end its commemoration ceremonies with a visit to the Colonial Exhibition^24^. The *Femmes de France* honored themselves with “privileged entries” issued by the Exhibition's commissioner. But, the inauguration day was postponed and a large number of U.F.F. members found themselves in front of closed doors of the designated pavilions.

“[They] were only able to do a complete tour outside the building, under lashing rain; but, although they had been hardened during the war, they were nevertheless greatly interested in the show and also in explanations kindly supplied by Mr. the administrator of the colonies who showed himself willing to monitor them”^25^.

The dynamism of the U.F.F. was so blatant that “they had no need (during the Colonial Exhibition) to keep in touch with their sister societies” (Boissier, 1985, p. 333). Madame Saint-René Taillandier (1865–1959), the philosopher Hippolyte Taine's niece and future chairwoman^26^, readily praised her own society:

“On this, our fiftieth anniversary, let us feel the strength of the link that binds us with one and another, in the present as in the past, to the chairwomen who have come after, to the one who after 40 years of “service-duty,” still generates today strength and tenderness over us” (Française, 1931, p. 6)^27^.

This call was far from the first U.F.F. claim asking the S.S.B.M. and the A.D.F. for collaboration and deployment of forces. Nurses were not exactly working with each other at world fairs but they had been through similar training. Like Dr. Duchaussoy at the beginning of the A.D.F., Dr. Boulimé had trained U.F.F. ladies^28^. But in the long run, U.F.F. trainees were given more esteem for organizing visitors' protection. Both this conversion and diversion between these two relief societies also lied in terms of their members. By the mid-1930s, the A.D.F. and the U.F.F. had a similar increase in their number of members (Pineau, 2006, p. 11–12), with the greatest prestige for the U.F.F. in International Exhibitions held in France. In this rival situation, the S.S.B.M. stood back, although it had over 140,000 supporters after the 1937 Paris World Fair.

## Gaining a World-Wide Dimension

There were many reasons why the U.F.F. had a prominent place in International Exhibitions. Its awards and professionalism can be highlighted here. It started at the 1931 Colonial Exhibition when the U.F.F. received two awards. The first was a Grand Prize^29^ obtained under the Metropolitan section in charge of developing exchanges with the colonies. Testimonies from colonizers relating, for example, the disappearance of small pox in Madagascar thanks to the U.F.F. endeavor, especially generated public interest^30^. The second award was no other than the gold medal which was presented by the Commissioner General to the U.F.F. for being the best exhibitor at the Colonial Exhibition^31^.

The U.F.F. International fame and recognition also lied in providing care at the Aid-Post. Between 15 May and 25 November, 1931, 82 nurses had treated 5,114 visitors and organizers

“…which gives an average of 28 persons per day. Amongst the diagnostics noted on patient records we can see several serious falls resulting in spinal fractures, fractures of limbs, deep wounds with severing of the arteries, dislocations; a large number of people passing out, two fatal heart attacks, some epileptic fits, one renal colic attack, one acute appendicitis, an alcoholic coma, a pulmonary edema, all necessitating evacuation to neighboring hospitals or calling an ambulance to drive the injured to their homes”^32^.

But those U.F.F. activities at Aid-Posts of the Colonial Exhibition were considered as quite secondary. The society's major concern was to make itself known, and promote its image on an International site. Therefore, as early as 1935, the U.F.F. launched an appeal to metropolitan committees in Algeria, Morocco, Tunisia, in countries under mandate as well as foreign countries to kindly send them “as soon as possible and ex gratia, pretty views of the outsides or insides of their establishments”^33^. Nursing schools, dispensaries, sanatoriums, preventoria^34^, open air schools, consultations for prenatal and the newly born, creches, nurseries, formula milk^35^, holiday camps were thus required to prepare for the International Exhibition of Arts and Technology in Modern Life which was to be held in Paris 2 years later.

When visitors arrived at the Palais de Chaillot in May 1937, Spain was in the middle of civil war (see also Vilar, 1994; Bloch, 2014). The I.C.R.C. had been working actively with victims, prisoners and their families and concentrated in collecting money for the Spanish Red Cross^36^. However, French Red Cross Societies were to organize, attend and participate in the International Exhibition but with no mention of this in the I.C.R.C. bulletin. It was left to an unknown paid^37^ nurse and assistant manageress of the U.F.F. Mme. Jobert-Dalligny, to report. In office while there was a transfer of power between two U.F.F. chairwomen, Mme. Bardier-Hugo and Mme. Saint-René Taillandier (Pineau, 2006, p. 12), Mme. Jobert-Dalligny was probably an Infirmière-Hospitalière (a Hospital nurse with a state diploma)^38^. At a time when the history of professionalization of nursing was in progress with trainees in Léonie Chaptal's *Maison-Ecole* (a School-Home) divided between first class nurses for curing and second class nurses for caring (Belliard, 2009, p. 191; see as well Belliard, 2017), that meant Mme. Jobert-Dalligny had attended one of the five U.F.F. nursing schools (Ligue des Sociétés de la Croix-Rouge, 1928, p. 27). In 1889 courses concentrated on anatomy, surgery, symptomatology, fever, and other functional disorders^39^. She presumably knew more than an upper class amateur nurse with a *certificat d'auxiliaire* (paramedic certificate) that did:

“… not confer the right to cure, [but] knowing effective gesturing in the case of an accident…[It was] mainly intended for all women who could not devote the proper time for studying nursing and yet who wanted to be helpful and acquired elementary knowledge in hygiene” (Pineau, 2006, p. 49).

Although it was quite difficult to state precisely the content of Mme. Jobert-Dalligny's training at the U.F.F., she was definitively an experienced nurse, having spent some time in the Rabat Health Center (Chavenon, 2010, p. 165). Her office duty, on the contrary, indicated a lot on her background. As an assistant manageress at the U.F.F., Mme. Jobert-Dalligny might be called to replace the Head. Therefore, she knew how to organize meetings when needed, that is to say at least every 2 months, and draft reports. She also knew how to be part of the Personnel Committee where she sat with six other members. Her duty was to inquire on efficient employment in the society, to monitor nursing students during their training period, to handle relief and family relationships and to enlist auxiliary helpers^40^.

With such responsibilities, Mme. Jobert-Dalligny became the natural U.F.F. spokesperson at the 1937 International Exhibition and filled the “space for women.” She stood at a time when women's suffrage was consistently rejected and when there was an “ambiguous reflections of the question of women's rights in France (Reynold, 2017). According to Mme. Jobert-Dalligny the Red Cross was fulfilling the well-being of mankind in presenting its general activities and providing medical care^41^. Her report was not just a professional witness account of what took place. It pinpointed women's polyvalent two-fold nature. They were able, at the same time, to exhibit and also to care. However, the 1937 International Exhibition exhorted their exhibiting activities. They preferred to bear witness to their hedonic activities rather than to the caring ones. They also were just as much attached to the spirit of competition as to the prizes or in-kind donations that they received to thank them for the great care they gave to the injured. The U.F.F. therefore seemed to be turning more toward patriotism than toward hygiene matters. That war more profitable and more promising in a world-wide approach.

This 1937 experience at the World Fair stood against the already existing collaboration among International Red Cross Societies where professionalism was important. The Anglo-French-American Hospital, in Neuilly-sur-Seine, for example, was run by a Red Cross “… lady versed not only in nursing but in the practical details of organization…”^42^. Her duties, similar to those of Mme. Jobert-Dalligny, were to build up a network and not separate the nations.

But, in an International Exhibition context, the U.F.F. tended to isolate itself and opposed the worlds of medicine and politics. Women in this French Red Cross society positioned themselves primarily as distinguished guests. They were there more for the show than as a reference.

Universal or International Exhibitions that took place in Paris had definitively a different impact on Women in French Red Cross Societies. If it was too early for Red Cross Ladies to invest in the first Universal Exhibition, the 1867 World Fair, held in Paris, was an opportunity to organize and structure the newly born International Relief Movement. But, the 1900 Universal Exhibition was an obstacle and a competitive place where French Red Cross Societies argued about the space they were allotted. The 1931 Colonial Exhibition worsened the relationship among French relief societies to the extent that only U.F.F. women were heard by the I.C.R.C. and held the front stage. In the history of the Red Cross Societies, the French case was peculiar in a sense that there were competing objectives. These were forecasting to a certain extent the 1940 legislation signed by the Maréchal Pétain enforcing a single French Red Cross society (Rouge, 1963, p. 18; Chauvy, 2000, p. 115–123; Bras, 2002, p. 62–66)^43^. The persistent predominance of the U.F.F. women again at the 1937 International Exhibition was not just a matter of hovering in their own space, but a question of collaboration that would be imposed in the near future. The framework reached its turning point by 1951 at Lille's World Fair. These early International or Universal Exhibitions served as a launching pad for women's national philanthropic unity.

## Author Contributions

The author confirms being the sole contributor of this work and has approved it for publication.

### Conflict of Interest Statement

The author declares that the research was conducted in the absence of any commercial or financial relationships that could be construed as a potential conflict of interest.

## Footnotes

^1^For a somewhat general biography of Henry Dunant (see Chaponnière, 2010).

^2^On the early women's devotion (cf. Guillermand, 1988).

^3^Philanthropy has been partly defined as such in *Trésor de la Langue Française*, 1986, Paris, Centre National de la Recherche Scientifique.

^4^https://www.icrc.org/fre/assets/files/other/554-557_livres.pdf (accessed March 17, 2015). *On the relations between Gustave Moynier and Henry Dunant* (cf. Bennett, 1983).

^5^These representatives came from all over Europe and also from several American States (cf. Boissier, 1985, p. 225; see also Française, 1998, p. 10–11).

^6^*La France Charitable et Prévoyante*, 1899, Paris, Plon.

^7^On the different chairwomen of the S.S.B.M. Ladies Committee see page 2 of General meetings from 1864 to 1937.

^8^This adjective was used by Gustave Moynier in his memoir on the founding of the Red Cross and handed out to the International Committee in 1903.

^9^http://www.icrc.org/fre/resources/documents/misc/5fzf8w.htm (accessed March 19, 2015).

^10^*Bulletin de l'Association des Dames Françaises*, n°6, 19th year, May 1905, p. 123.

^11^*Bulletin de l'Association des Dames Françaises*, n°6, 19th year, May 1905, p. 118.

^12^Obituary notice signed by Dr. Duchaussoy, in *Bulletin de l'Association des Dames Françaises*, n° 3, 25th year, February 1911, p. 65–70.

^13^On the programmes led by women in the medical field (see Diebolt, 1984).

^14^*Bulletin International des Sociétés de la Croix-Rouge*, n° 124, 31st year, October 1900, p. 237.

^15^Mme. Bardier-Hugo's speech, U.F.F. chairwoman, in *Bulletin de l'Association des Dames Françaises*, n° 5, 12th year, May-June 1931, p. 269.

^16^Cf. the 1900 International Congress on women's rights and conditions—Congrès international de la condition et des droits des femmes, 1901, *Congrès international de la condition et des droits des femmes, tenu les 5, 6, 7 et 8 septembre 1900 à l'Exposition universelle, au Palais de l'économie sociale et des congrès*: *questions économiques, morales et sociales, éducation, législation: droit privé, droit public*, Paris, Imprimerie des arts et manufactures (see also Durand, 1901; Rasmussen, 1989).

^17^*Bulletin International des Sociétés de la Croix-Rouge*, n° 123, 31st year, July 1900, p. 177.

^18^*Bulletin International des Sociétés de la Croix-Rouge*, n° 124, 31st year, October 1900, p. 225.

^19^*Revue de l'Union des Femmes de France*, n° 9, 12th year, October 1931, p. 399.

^20^*Bulletin International des Sociétés de la Croix-Rouge*, n° 350, part LXII, October 1931, p. 891.

^21^See also *Revue de l'Union des Femmes de France*, n° 9, 12th year, October 1931, p. 400.

^22^*Revue de l'Union des Femmes de France*, n° 9, 12th year, October 1931, p. 401.

^23^It was mentioned as early as February; cf. *Bulletin International des Sociétés de la Croix-Rouge*, n° 342, part LXII, February 1931, p. 162.

^24^*Revue de l'Union des Femmes de France*, n° 5, 12th year, May 1931, p. 283.

^25^*Revue de l'Union des Femmes de France*, n° 5, 12th year, May 1931, p. 284.

^26^*Revue de l'Union des Femmes de France*, n° 5, 19th year, October 1938.

^27^*Union des Femmes de France – Cinquantenaire 1881–1931*, 1931, Paris, Croix-Rouge Française: 6.

^28^Mme. Bardier-Hugo's speech, U.F.F. chairwoman, in *Revue de l'Union des Femmes de France*, n° 5, 12th year, May–June 1931, p. 269.

^29^*Revue de l'Union des Femmes de France*, n° 11, 12th year, December 1931, p. 463.

^30^*Revue de l'Union des Femmes de France*, n° 10, 12th year, November 1931, p. 454.

^31^*Revue de l'Union des Femmes de France*, n° 11, 12th year, December 1931, p. 466.

^32^*Revue de l'Union des Femmes de France*, n° 11, 12th year, December 1931, p. 470.

^33^*Revue de l'Union des Femmes de France*, n° 9, 15th year, October 1935, p. 287.

^34^Singular for “preventorium.” It was “preventive” a sanatorium where patients suffered from a first tuberculosis infection and had no risk of contagion. cf. http://www.larousse.fr/dictionnaires/francais/pr%C3%A9ventorium/63872 (accessed March 14, 2015).

^35^Milk charity for mothers in the physical or social inability to feed their children with good quality milk (cf. Julien, 1997).

^36^*Bulletin International des Sociétés de la Croix-Rouge*, n° 413, part LXVIII, January 1937, p. 92–97.

^37^From 1922 all nurses were paid. This came along with the establishment of a state diploma (cf. Pineau, 2006, p. 15; Chavenon, 2010, p. 105).

^38^On the different kind of nurses i.e., assistant nurses with an elementary, first or second degree, visiting nurses for tuberculosis and pediatric, and hospital nurses (cf. Ligue des Sociétés de la Croix-Rouge, 1928, p. 26–27). See also Ecole d'infirmières de la Croix-Rouge française, no date, *Société de secours aux blesses militaires – Hôpital-Ecole*, Paris: 3. See as well Pineau (2006, p. 15) or again Diebolt and Fouché (2011).

^39^Union des Femmes de France, 1889–1890, Personnel Committee, 10 pages handwritten notes on “Cours de médecine élémentaire donnés à l'Union des Femmes de France pour les membres auxiliaires: notions générales d'anatomie – pansement, petite chirurgie – symptomatologie – embolie, fièvre, troubles fonctionnels.”

^40^Union des Femmes de France, 1889–1890, Personnel Committee, 10 pages handwritten notes on “*Composition et fonctionnement de la Commission*.”

^41^*Revue de l'Union des Femmes de France*, n° 1, 19th year, January 1938, p. 3–5.

^42^British Committee sitting at the London Homeopathic Hospital, no date, *The Anglo-French-American Hospital: an account of the work carried on under Homeopathic Auspices during 1915–1916 at the Hôpital Militaire Auxiliaire*, n° 307, Neuilly-sur-Seine, in conjunction with the French Red Cross Society, p. 18–19.

^43^See also the account of Madame la Maréchal Lyautey's niece, Jacqueline Mallet, *Du joug à la délivrance, Paris 1940–1945*. Paris: Chantenay, 13.
